# Off-Stoichiometry Thiol-Ene (OSTE) Micro Mushroom Forest: A Superhydrophobic Substrate

**DOI:** 10.3390/mi15091088

**Published:** 2024-08-28

**Authors:** Haonan Li, Muyang Zhang, Yeqian Liu, Shangneng Yu, Xionghui Li, Zejingqiu Chen, Zitao Feng, Jie Zhou, Qinghao He, Xinyi Chen, Huiru Zhang, Jiaen Zhang, Xingwei Zhang, Weijin Guo

**Affiliations:** 1 Department of Electrical Engineering, Shantou University, Shantou 515063, China; 2 Department of Biomedical Engineering, Shantou University, Shantou 515063, China; 3 Department of Biology, Shantou University, Shantou 515063, China; 4 Guangdong University Research Findings Commercialization Center, Foshan 528000, China; 5 Department of Mechanical Engineering, Shantou University, Shantou 515063, China

**Keywords:** OSTE, superhydrophobic, backside lithography, micro mushroom, surface topology, diffused UV light

## Abstract

Superhydrophobic surfaces have been used in various fields of engineering due to their resistance to corrosion and fouling and their ability to control fluid movement. Traditionally, superhydrophobic surfaces are fabricated via chemical methods of changing the surface energy or mechanical methods of controlling the surface topology. Many of the conventional mechanical methods use a top-to-bottom scheme to control the surface topolopy. Here, we develop a novel fabrication method of superhydrophobic substrates using a bottom-to-top scheme via polymer OSTE, which is a prototyping polymer material developed for the fabrication of microchips due to its superior photocuring ability, mechanical properties, and surface modification ability. We fabricate a superhydrophobic substrate by OSTE–OSTE micro mushroom forest via a two-step lithography process. At first, we fabricate an OSTE pillar forest as the mushroom stems; then, we fabricate the mushroom heads via backside lithography with diffused UV light. Such topology and surface properties of OSTE render these structures superhydrophobic, with water droplets reaching a contact angle of 152.9 ± 0.2°, a sliding angle of 4.1°, and a contact angle hysteresis of less than 0.5°. These characteristics indicate the promising potential of this substrate for superhydrophobic applications.

## 1. Introduction

Usually, when the contact angle of a water droplet on a surface is greater than 150 degrees in nature, we can call such surface a superhydrophobic surface [1]. There are many superhydrophobic surfaces in nature, such as the lotus leaf and leg of Aquarium paludum Fabricius. Superhydrophobic substrates have been applied in various fields of engineering, such as self-cleaning, anti-corrosion, oil or water separation, anti-icing, antifogging, and antifouling [2]. People have been working on various methods of fabricating superhydrophobic substrates [3,4]. In general, there are two ways to fabricate superhydrophobic substrates. One is via chemical methods for surface energy modification, of which the purpose is to decrease the surface energy of the substrate, such as superhydrophobic spray. Yu et al. developed a hydrophobic spray based on silica particles [5]. Xie et al. provided a superamphiphobic coating surface with bionic microstucture via FPU/PMMA mixture [6]. Asadollahi et al. used an atmospheric pressure plasma jet to develop an organosilicon-based superhydrophobic coating [7]. The fabrication of superhydrophobic coatings through electrodeposition on metals that are readily oxidizable is also a typical strategy [8]. Another method for the fabrication of superhydrophobic substrates is through manipulation of the surface topology using mechanical methods. For researchers in the field of microsystems, there is an interest in changing the surface topology to construct a superhydrophobic surface. In contrast to surface energy modification, the manipulation of surface topography involves the design of composite microstructures on the substrate, thereby altering the surface properties of the substrate, which can improve the stability and durability of the substrate at the same time. Etching has often been used to fabricate superhydrophobic surfaces by metal, such as aluminium and titanium [9,10,11]. The fabrication of superhydrophobic substrates by designing silica templates and utilizing soft lithography has also been demonstrated to be an effective solution [12]. In addition, Wang et al. created an integrated topological superhydrophobic structure on aluminium alloy via femtosecond laser ablation [13].

Among various surface topologies, there is a typical kind for superhydrophobic substrates: the mushroom-like microstructure. This structure exhibits re-entrant characteristics and generally shows favorable superhydrophobic properties. Researchers have used different methods for fabrication of these mushroom-like microstructures. Liu et al. fabricated a mushroom-like superhydrophobic microstructure via the etching of a silicon substrate [14]. Cumont et al. prepared nickel micro mushroom structures using UV-assisted nanoimprint lithography and tested their antimicrobial properties [15]. Nevertheless, the fabrication procedures of both silicon etching and nanoimprinting are complex. With the fast development of 3D printing technology, many researchers have employed 3D printing for the fabrication of mushroom-like superhydrophobic microstructures [16,17]. However, the precision and working principle of the 3D printer impose limitations on the surface properties of microstructures, including surface roughness, which in turn affects the surface properties of the substrate. In short, the fabrication of superhydrophobic substrates with micro mushroom structures is a highly active research area, but the existing techniques still have some limitations. The majority of these techniques, including etching and laser burnishing, employ a top-to-bottom fabrication process, which can increase the overall time required for fabrication. Additionally, the need for specialized instruments, the presence of cumbersome procedures, and the generation of non-negligible surface roughness, all cause inconveniences in the fabrication process and prevent further applications of these techniques.

In this work, we propose a novel bottom-to-top strategy for the fabrication of a superhydrophobic substrate using polymer OSTE–OSTE micro mushroom forest. OSTE is formed by thiol monomers and ene monomers with an off-stoichiometry mix ratio and cured via click chemistry under UV irradiation. It is a transparent photocurable polymer that exhibits favorable mechanical properties and has reactive surface groups upon curing [18]. OSTE is compatible with a multitude of traditional microfabrication techniques, including casting, reaction injection molding, photolithography, and micromachining. OSTE has been used in the fabrication of a wide variety of microfluidic chips [19,20,21,22,23]. Moreover, OSTE photolithography is highly adaptable and can be employed to fabricate intricate microstructures [24]. Recently, a fabrication method utilizing OSTE backside lithography with a light diffuser has been developed for the manufacturing of convex microstructures [25]. We plan to fabricate OSTE micro mushroom forest using photolithography of OSTE.

We fabricate OSTE micro mushroom forest via a two-step lithography. The initial step is to construct an OSTE pillar forest on a flat substrate. Subsequently, a light diffuser is employed to disperse the parallel UV light. OSTE pillars are utilized as optical waveguides to reflect the diffused UV light. Finally, OSTE is cured on top of the pillars to form the mushroom heads via backside lithography [25,26]. Contact angle and sliding angle tests are used to characterize the surface properties of the OSTE micro mushroom forest.

## 2. Materials and Methods

Chrome glass mask is from Jixian Opto-electronic (Shenzhen, China). Isopropyl alcohol (IPA) and gelatin are from Macklin (Shanghai, China). Propylene glycol monomethyl ether acetate (PGMEA) is from Aladdin (Shanghai, China). Black dye is from Fleur Couleur (Zhejiang, China). Diffusing glass is from Edmund Optics (75 mm Diameter Opal Diffusing Glass, Barrington, NJ, USA). UV lithography machine is from the Institute of Optics and Electronics, Chinese Academy of Sciences (URE-2000/35AL, Chengdu, China). Polymer OSTE is prepared according to a previous work [18].

**Figure 1:**
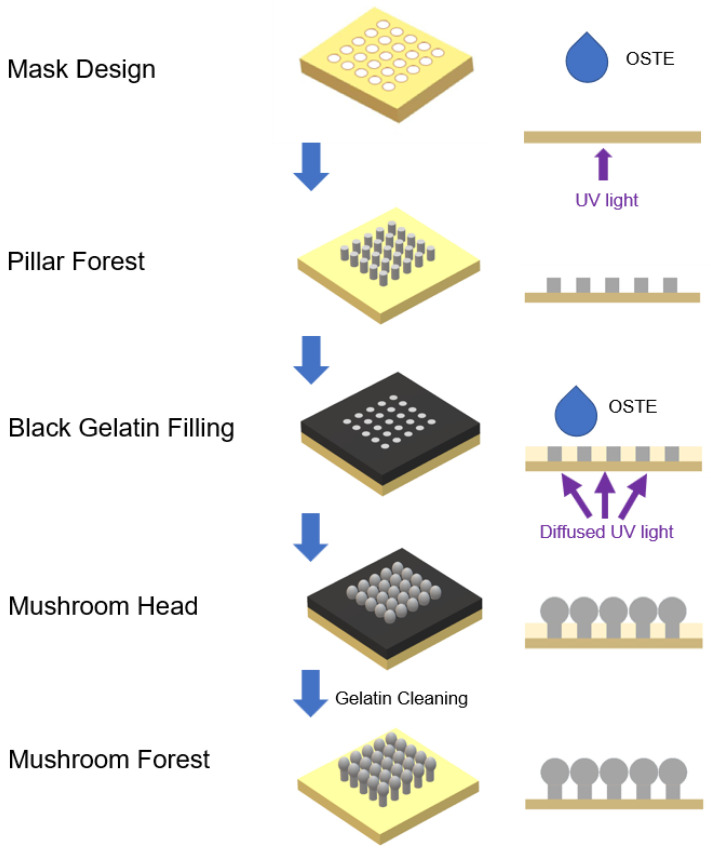
Schematic diagram of the fabrication of OSTE micro mushroom forest. At first, we fabricate the OSTE pillar forest on a flat substrate. Then, we use black gelatin solution to fill the space between pillars under capillary action and solidify gelatin. After that, diffused UV light is reflected through pillars and cures the OSTE contacting the pillar top, thereafter forming the OSTE micro mushroom forest. This diagram is not to scale.

**Figure 2:**
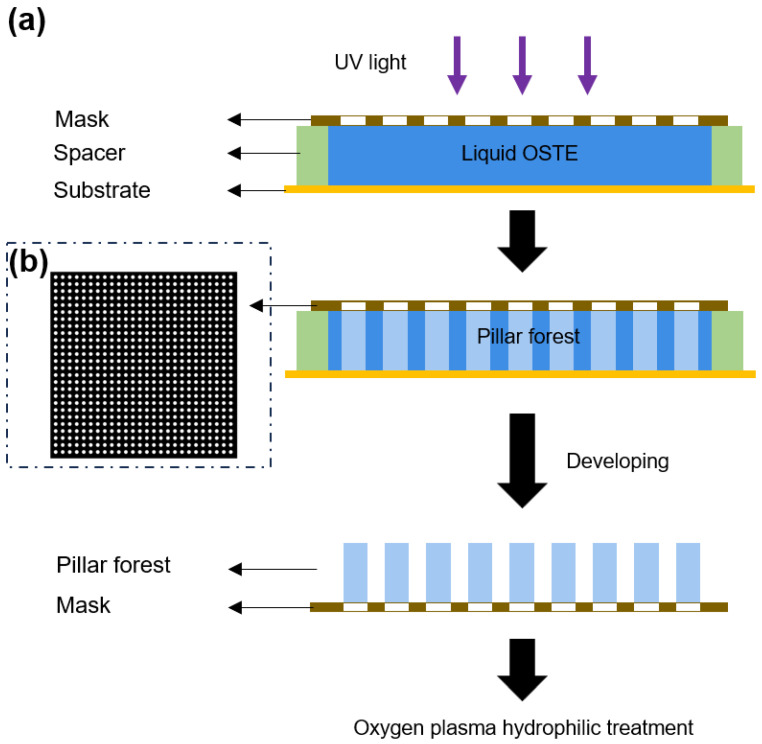
(**a**) The fabrication procedures of OSTE pillar forest in cross-section view. The pillars are in an orthogonal arrangement. (**b**) The design of the chrome glass mask.

The fabrication of the OSTE micro mushroom forest can be divided into two main parts: OSTE pillar forest and OSTE mushroom heads. Figure 1 shows a 3D schematic diagram of the entire fabrication process. The first step is the fabrication of OSTE pillar forest, and Figure 2a shows a cross-section view of the fabrication process of OSTE pillar forest. CAD software (AutoCAD 2022, Autodesk, San Rafael, CA, USA) is employed to design the chrome glass mask, and the mask design is shown in Figure 2b. Subsequently, the mask is placed in contact with uncured OSTE, and a plastic spacer of specific thickness is added between the substrate and the mask to define the height of the pillars. After UV exposure, the chromium mask is removed and developed using PGMEA, followed by cleaning with IPA to obtain the pillar forest. Then, the pillar forest is subjected to an oxygen plasma treatment.

**Figure 3:**
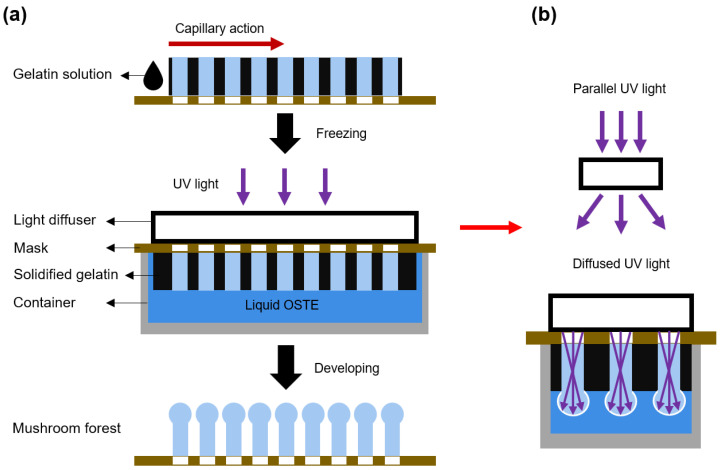
(**a**) The cross-section view of the fabrication process of the OSTE micro mushroom forest. (**b**) The working principle of the light diffuser and the fabrication of the mushroom head: diffused UV light is reflected in the pillars and emitted from the pillar top, curing a spherical microstructure.

The second step is the fabrication of mushroom heads, and a cross-section view is illustrated in Figure 3a. Gelatin solution (10% in DI water) with 1% black dye is prepared and dropped onto the pillar forest. The black gelatin solution is then drawn into the interstices of the pillars via capillary action. Subsequently, the gelatin is allowed to solidify at 4 °C. After that, the OSTE pillar forest with solidified gelatin is put in contact with uncured OSTE and then covered with a light diffuser. Subsequently, the pillars are subjected to UV light curing, development, and post-curing. As the light diffuser transforms the parallel UV light into diffused UV light, the diffused UV light is reflected in the pillars, thereby curing the OSTE on top of the pillars and forming spherical microstructures. The working principle of the UV light diffuser and the fabrication of the mushroom heads are shown in Figure 3b. Ultimately, the residual gelatin is removed by washing of water of 37 °C, resulting in the final OSTE micro mushroom forest.

The experimental images are captured by a stereomicroscope (Leica M205C, Wetzlar, Germany). Contact angle measurements are conducted via a contact angle measurement machine (Sindin SDC-200S, Dongguan, China).

## 3. Results and Discussion

**Figure 4:**
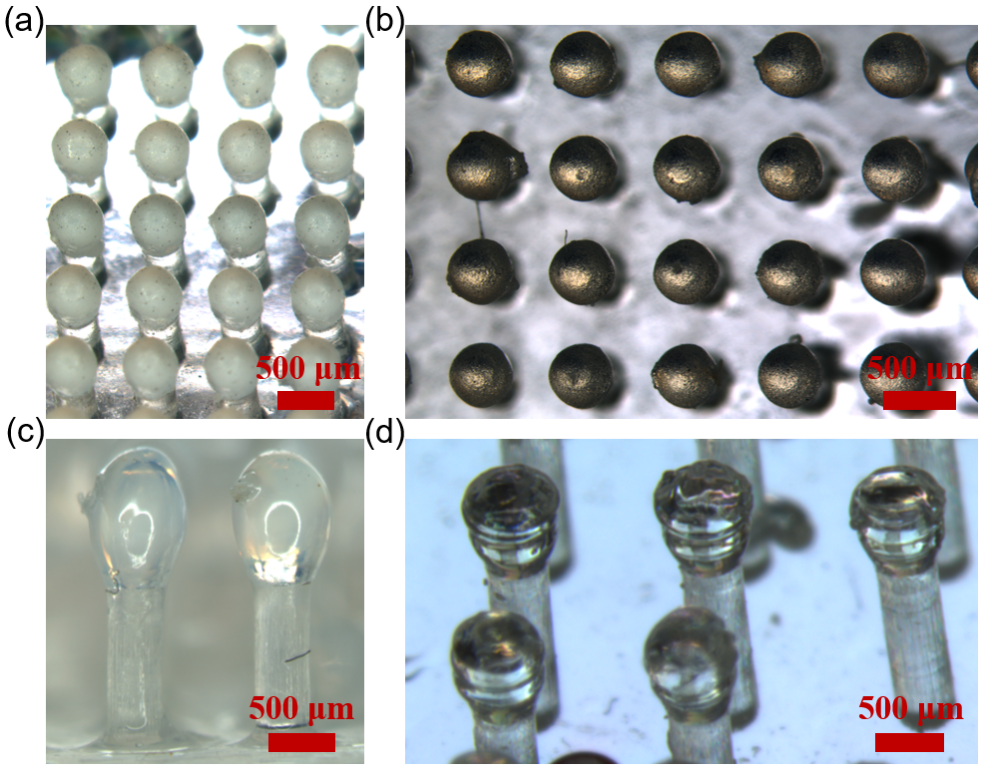
(**a**,**b**) OSTE micro mushroom forest with a pillar diameter of 300 μm and a distance of 400 μm. (**c**) OSTE micro mushroom forest with a pillar diameter of 400 μm and a distance of 600 μm. (**d**) OSTE micro mushroom forest with a pillar diameter of 400 μm and a distance of 1000 μm. The difference in brightness of the images is due to different imaging angle, illumination intensity, and exposure time.

We successfully fabricate OSTE micro mushroom forest of different dimensions with pillar diameters ranging from 100 μm to 400 μm. As shown in Figure 4, the diameters of the pillars are 300 μm (Figure 4a,b) and 400 μm (Figure 4c,d). It is clearly shown that there is a spherical head on the straight pillars.

**Figure 5:**
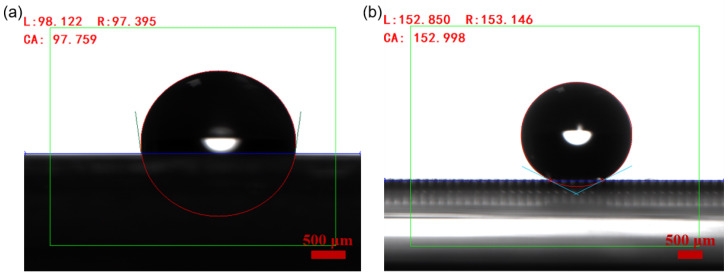
(**a**) The contact angle of a water droplet on a flat OSTE substrate. (**b**) The contact angle of a water droplet on the OSTE micro mushroom forest (with a pillar diameter of 100 μm and a distance of 100 μm), which is 152.9 ± 0.2°. The volume of the water droplet in both images is 5.0 μL.

**Figure 6:**
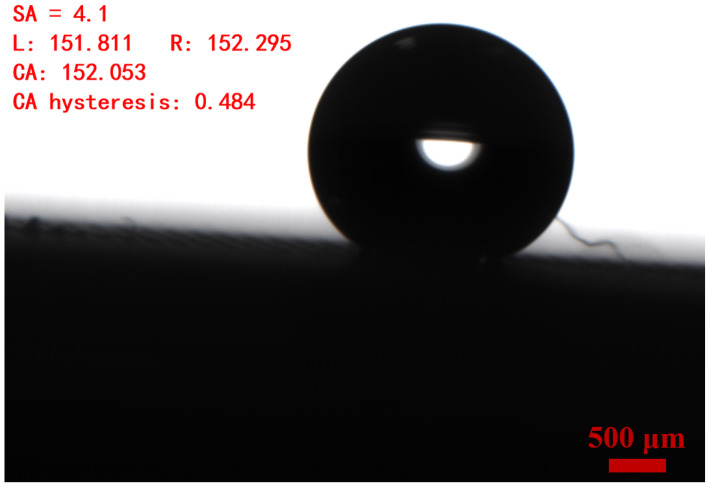
The sliding angle of a water droplet with a volume of 5.0 μL on OSTE micro mushroom forest (with a pillar diameter of 100 μm and a distance of 100 μm) is evaluated, with a sliding angle of 4.1° and a contact angle hysteresis of 0.484°.

**Figure 7:**
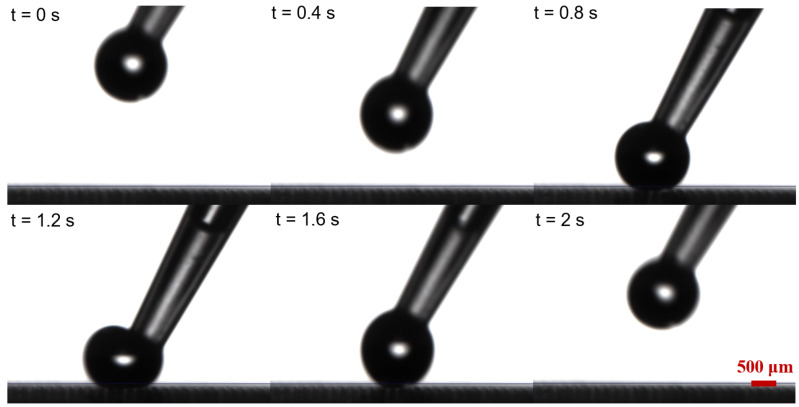
A series of images that show the low adhesion of water droplets on OSTE micro mushroom forest (with a pillar diameter of 100 μm and a distance of 100 μm). The volume of the water droplet is 5.0 μL.

**Figure 8:**
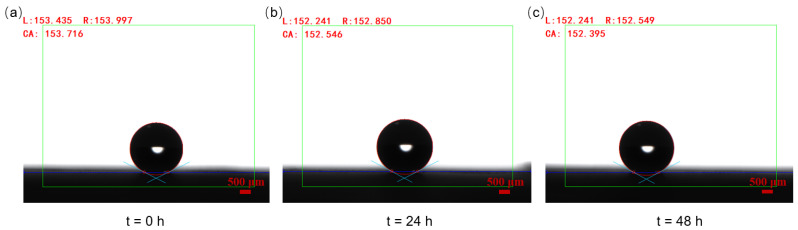
(**a**–**c**) The images illustrate the water droplet contact angle tests conducted on OSTE micro mushroom forest (with a pillar diameter of 100 μm and a distance of 50 μm) over a period of time after fabrication. The volume of the water droplet is 5.0 μL.

The contact angle of water droplets on OSTE micro mushroom forest exceeds 150°, with a contact angle hysteresis of less than 0.5° and a sliding angle of less than 5°, as shown in Figure 5 and Figure 6, indicating the superhydrophobicity of the substrate. Compared to a flat OSTE substrate, the enhancement of the micro mushroom forest in terms of hydrophobicity is significant, as shown in Figure 5. In addition, we conduct an adhesion test of water droplets on this substrate, and Figure 7 shows the low adhesion of OSTE micro mushroom forest to water droplets (also shown in Video S1). Furthermore, a series of stability tests have been conducted on OSTE micro mushroom forest, as illustrated in Figure 8. OSTE micro mushroom structure is shown to remain superhydrophobic with increasing storage time. These tests specifically check the alteration of the superhydrophobicity of OSTE micro mushroom forest over time after fabrication. In addition, the surface properties of polymer OSTE is quite stable in air environment, which could facilitate the storage of this substrate.

This fabrication method has a high design flexibility in terms of the dimensions of the microstrucrures, including the diameter and height of the pillars. This two-step lithography method allows for the straightforward fabrication of a large-area OSTE micro mushroom forest in a short period of time. This fabrication method is compatible with a mask size of 5 inch × 5 inch, and the whole fabrication process takes less than 30 min. In theory, the area of OSTE micro mushroom forest that can be fabricated via this method is determined by the size of UV light source. Therefore, it is possible to fabricate a larger OSTE micro mushroom forest if the size of UV light source is big enough. In addition, the area of OSTE micro mushroom forest has an insignificant influence on the speed of fabrication. Moreover, since OSTE has adjustable mechanical properties, including the E-modulus [18], the robustness of OSTE micro mushroom forest can also be tuned for different application scenarios. We believe that this method has a significant potential in the field of microsystems, such as in the fabrication of substrates for liquid motion control and antibacterium [27].

## 4. Conclusions and Outlook

A novel bottom-to-top strategy is developed for the fabrication of a superhydrophobic substrate by polymer OSTE–OSTE micro mushroom forest. This method is more efficient and straightforward than traditional microfabrication methods, and it allows for the fabrication of large-area superhydrophobic substrates in a relatively short period of time. Furthermore, the method is flexible since the dimensions of OSTE micro mushroom forest can be easily modified by adjusting the mask design. OSTE micro mushroom forest exhibits excellent superhydrophobic properties and low adhesion properties, with a contact angle of 152.9 ± 0.2°, a sliding angle of 4.1°, and a contact angle hysteresis of less than 0.5°. As to the future outlook, we believe that by scaling down the mask design and improving the fabrication process, we will eventually be able to fabricate micro mushroom structures with pillar diameters of tens of micrometers for further enhancement of superhydrophobic properties. It is also possible to fabricate tilted micro mushroom structures through this method, which can be used for directed droplet bouncing. Furthermore, we envision that this method should be compatible with certain other photocurable polymers. This approach has a significant potential for the research of superhydrophobic substrates in the near future.

## Data Availability

The original contributions presented in the study are included in the article, further inquiries are available per request addressed to guoweijin@stu.edu.cn.

## Supplementary Materials

The following are available online at https://www.mdpi.com/article/10.3390/mi15091088/s1, Video S1: Adhesion test.
